# Flowing toward toughness: serial mediation of flow and mental toughness in gamified XR soccer instruction

**DOI:** 10.3389/fpsyg.2026.1731891

**Published:** 2026-02-23

**Authors:** Chao Li, Daniel Memmert, Guangliang Sang

**Affiliations:** 1 Faculty of Education Science & Technology, Universiti Teknologi Malaysia, Johor Bahru, Malaysia; 2 Institute of Exercise Training and Sport Informatics, German Sport University Cologne, Cologne, Germany; 3 College of Arts and Physical Education, Kangnam University, Yongin, Republic of Korea; 4 School of Engineering, Korea National University of Transportation, Chungju, Republic of Korea

**Keywords:** extended reality (XR), flow experience, gamification, learning engagement, mental toughness, serial mediation, sport attachment

## Abstract

Extended reality (XR) platforms offer immersive, gamified teaching methods. However, the psychological mechanisms by which XR promotes effective sports learning remain under-explored. Drawing on self-determination theory and challenge-skill balance theory, this study proposes and tests a serial mediation model that links learners’ evaluations of game-based design and their own self-regulatory abilities to soccer learning engagement and subsequent sport attachment. The model’s serial structure is based on the temporal progression from situational experience to trait development: it posits that the immediate, optimal state of “flow” during a session acts as a mastery-building catalyst that eventually consolidates into enduring “mental toughness.” Partial least squares-structural equation modeling (PLS-SEM) was used to analyze survey data from 620 higher education students enrolled in XR-based soccer courses. The results show that perceived game elements do not directly enhance learning effort. Rather, their influence is fully transmitted sequentially through the building of flow and subsequent mental toughness. Additionally, perceived self-control has a significant direct effect on learning effort and an indirect effect through mental toughness and the flow-toughness chain, but not solely through flow. Both situational flow and resilient mental states independently enhance active participation, which in turn promotes long-term sport attachment. This model highlights the importance of interweaving emotional and cognitive states in XR sports contexts to mobilize learning efforts. The findings refine gamification-in-education theory by demonstrating how transient optimal experiences accumulate into lasting psychological resources.

## 1 Introduction

Extended reality (XR), encompassing virtual reality (VR), augmented reality (AR), and mixed reality (MR), has advanced rapidly in educational contexts, driven by technological progress and increasing recognition of its capacity to enhance learning (Burke et al., 2025). XR is an inclusive term. It encompasses a variety of immersive technologies and digital environments that are designed for representing and projecting data (Wang and Ahn, 2024). New intelligent technologies represented by XR have become the core driving force of the Fourth Industrial Revolution. Their integrated applications in the field of education are also promoting the continuous transformation of teaching toward gamification (Yang, 2024; Feng et al., 2025). Technological advancements and the growing popularity of 360° cameras and affordable XR devices have paved the way for XR’s educational applications (Cotterill, 2018). Meantime, recent empirical work shows that XR-based interventions do not consistently outperform well-designed non-immersive instruction. Specifically, effect sizes vary across domains, learner characteristics, and task designs (Han et al., 2025; Stanney et al., 2023).

In sports, coaches and educators are continuously integrating new technologies into training to improve learners’ performance (Ben Mahfoudh and Zoudji, 2025). For instance, Stinson and Bowman (2014) examined the potential of XR for training soccer goalkeepers in high-pressure situations using simulated penalty-kick training. Within soccer instruction, XR supports student-centered pedagogy by simulating decision-making scenarios, reproducing perceptual and cognitive demands, dynamically calibrating task difficulty, and delivering diagnostic feedback. These mechanisms foster greater time on task, strategic engagement, and affective involvement (Radianti et al., 2020; Yang et al., 2025), thereby providing practical means to strengthen deliberate practice and improve learning outcomes. However, these simulations still abstract away from the full physical, social, and psychological complexity of real play. Poorly calibrated XR tasks may fragment learners’ focus across multiple sensory channels, thereby constraining their pedagogical effectiveness (Le Noury et al., 2022; Makransky et al., 2019).

Nevertheless, although gamified XR designs are increasingly embedded within curricula, the psychological pathways through which interactive affordances translate into sport attachment remain not yet sufficiently explained (Fortes et al., 2021). While “sports identity” describes how individuals define themselves through their athletic role, this study focuses on “sport attachment,” the enduring affective bond and psychological commitment to the sport, as the primary outcome of interest. Current research often emphasizes platform characteristics, such as presence and fidelity (Kristina et al., 2025; Reneker et al., 2020). However, these technical metrics offer limited insight into sustained involvement because they prioritize the realism of the simulation over the learner’s subjective psychological experience and the internalization of the activity. Previous studies show that while gamification can improve soccer learning outcomes, its effects are inconsistent and often depend on extrinsic incentive mechanisms rather than direct skill enhancement (Zeng et al., 2024).

Moreover, gamification carries several risks in educational contexts, including technical acceptance difficulties, heightened competitive pressure for some students, and the possibility that superficial game elements distract from core skill acquisition. From an institutional perspective, XR-based gamified instruction is also constrained by high financial and infrastructure demands, technical instability, and uneven teacher expertise, as well as by open questions regarding long-term transfer of learning. Furthermore, while physical education pedagogy has long recognized that repeated, meaningful engagement strengthens activity identity and that the immersion and rich experiences brought by XR enhance learning motivation, few XR studies have linked soccer learning engagement to sport attachment (Makransky and Petersen, 2021). The specific proximal states and capabilities that mediate the transfer of these effects to sustained engagement and sports attachment (key outcomes of continued participation beyond the classroom) have not been fully elucidated. Notably, the role of mental toughness, a learner’s ability to remain resilient and focused under the pressures of a gamified environment, remains under-explored as a cognitive-affective mediator in XR contexts.

To address this gap, this study integrates self-determination theory (SDT) and challenge-skill balance theory (a core component of flow theory) in a complementary framework. Specifically, SDT provides the motivational foundation, suggesting that perceived gamification (PG) and perceived self-control (PSC) satisfy the basic psychological needs for competence and autonomy. This satisfaction, in turn, facilitates the “flow” state described by the challenge-skill balance. We model how PG and PSC influence soccer learning engagement (SLE) and, ultimately, sport attachment (SA). These patterns align with meta-analytic findings regarding gamification, supportive environments, and the relationship between flow and performance (Zaric et al., 2021). Research based on SDT indicates that supporting autonomy, competence, and relatedness can enhance learners’ motivation and engagement (Howard et al., 2025; Ryan and Vansteenkiste, 2023). Converging evidence on flow experience indicates that optimal states arising from a challenge–skill balance are associated with superior performance and greater persistence in sport settings (Harris et al., 2023). Collectively, these findings delineate a pathway through which design features and learner appraisals energize effort via specific psychological states.

By treating PG and PSC as central mechanisms, this study argue that gamification serves as the external environment that triggers engagement, while self-control represents the internal capacity to regulate that engagement. Together, they bridge the gap between XR design features and the internal psychological states required for long-term sports adherence. Moreover, the model informs the integration of XR into soccer pedagogy. By monitoring learners’ capabilities and engagement, and by fostering mastery-oriented, meaningful experiences, XR-enabled soccer courses can enhance immediate learning outcomes, strengthen affective attachment to soccer, and promote the transfer of analogous motor-cognitive skills-aligning with higher education’s objective of supporting lifelong physical activity.

Accordingly, this study explores the following three questions:

1. How do perceived gamification and self-control translate into sustained learning engagement in XR-integrated physical education?
2. Given the potential interrelation of flow experience and mental toughness, rather than their independence, does repeated optimal experience in XR lead to a more resilient and regulated response pattern?
3. How is sports attachment established in technology-mediated physical education courses?

Clarifying these issues helps reconcile the mixed findings in the literature on extended reality and educational gamification. It also shifts the focus from platform-centered discourses to learner-centered process models.

## 2 Theoretical basis and research model

From the perspectives of integrated motivation theory and skill acquisition, this study explains how gamified, XR-based soccer instruction motivates students and enhances their engagement in sports. This study clarifies construct definitions, proposes directional hypotheses for direct, indirect, and serial effects, and integrates evidence from XR learning, gamification, sports psychology, and participation research. Ultimately, a research model is developed to illustrate how learning engagement can be enhanced and sports attachment promoted through the sequential mediation of flow-toughness in an XR environment. This model bridges the gap between transient experiential states (flow) and enduring psychological resources (mental toughness), providing a longitudinal view of learner development.

### 2.1 Theoretical basis

Extended reality (XR) provides opportunities for embodied practice and immediate feedback. However, the motivational impact of XR is not monolithic; it varies across its sub-technologies. Virtual reality (VR) offers high immersion that facilitates deep “presence” and flow by isolating the learner from distractions. In contrast, augmented reality (AR) and mixed reality (MR) allow for the integration of digital cues into the physical field, which may better support perceived self-control and the transfer of skills to real-world soccer environments (Cummings and Bailenson, 2016; Radianti et al., 2020). For skill learning such as soccer, XR can stage graded challenges, capture micro-performances, and surface analytic cues that would typically require expert guidance. These affordances shape motivational and self-regulatory processes that underwrite engagement. This study integrates self-determination theory with the challenge–skill balance framework to investigate the drivers of learning engagement in gamified XR for soccer instruction.

According self-determination theory (SDT), satisfaction of autonomy, competence, and relatedness needs fosters high-quality engagement (Ryan and Deci, 2000). Research also indicates that designing gamification elements based on the SDT can address students’ psychological needs for autonomy, competence, and relatedness. This enhances their engagement in learning and achievement of learning goals (Grabner-Hagen and Kingsley, 2023). In XR-supported soccer skill teaching, students are more likely to invest effort and effectively regulate their behavior when basic learning needs are supported through XR’s gamification elements (Deterding et al., 2011; Sailer et al., 2017). This study integrates self-determination theory (SDT) with the challenge–skill balance framework through a “motivation-experience-development” logic. SDT acts as the foundational layer: when XR design satisfies the needs for autonomy and competence, it creates the psychological readiness required for an optimal experience. According to SDT, satisfaction of these needs fosters high-quality engagement (Ryan and Deci, 2024). In this framework, perceived gamification and perceived self-control serve as the environmental and individual precursors that fulfill these needs.

Furthermore, flow theory posits that optimal experience arises when perceived challenges match perceived skills and is accompanied by clear goals and immediate feedback (Csikszentmihalyi, 1990; Engeser and Rheinberg, 2008). XR can help maintain this balance by algorithmically calibrating task difficulty and visualizing evolving skill states. Repeated flow episodes can enhance attentional control and promote appraisal of challenges as opportunities. These processes are closely related to mental toughness—perseverance, self-confidence, and emotional regulation under stress (Gucciardi et al., 2012). Furthermore, this study propose a “state-to-resource” transition to justify the flow-toughness link. While flow is a situational, transient state of optimal experience (Csikszentmihalyi, 1990), mental toughness is a more stable psychological resource (Gucciardi et al., 2012). Theoretically, repeated episodes of flow—where the learner successfully navigates high challenges—act as “mastery experiences.” These experiences gradually crystallize into mental toughness, as the learner internalizes the ability to maintain focus and emotional regulation under pressure.

Accordingly, this study models flow experience and mental toughness as interrelated mediators linking XR and instructional design features, together with self-regulatory capacities, to soccer learning engagement and, ultimately, sports attachment. Methodologically, sequential mediation can generate more precise mechanism testing and align the analysis with the logical teaching timeline (i.e., session-level experiences that precede the development of capabilities), thus providing feasible guidance for designing an XR-based soccer curriculum.

### 2.2 Driving factor analysis

Perceived gamification (PG) is defined as learners’ perception that design elements unique to games, such as points, levels, challenges, leaderboards, rewards, and narratives, constitute an XR course (Deterding et al., 2011; Hamari et al., 2014). Gamification has been proposed as a way to encourage people to engage in behaviors that are both personally and socially beneficial, such as exercising and pursuing an education. Gamification aims to motivate users to perform tasks promoted by the service (Deterding et al., 2011; Huotari and Hamari, 2012). This can be achieved by providing the affordances of a gamified experience and making the target activity more appealing. Previous research has shown that incorporating gamification elements that meet students’ basic psychological needs improves their sports skill performance, learning behavior, and learning engagement (Fernandez-Rio et al., 2020), particularly when clear goals are set and conditional feedback is provided (Sailer et al., 2017). They can also diagnose learning progress and competence. However, PG may also generate “extraneous load,” which will not automatically translate into greater effort unless transformed into an optimal experience or toughness beliefs (Hsia et al., 2025). Gamification learning theory predicts that these elements indirectly influence learning outcomes by shaping antecedent states (e.g., motivation, attention, and flow), thereby driving learning behaviors (Thomas and Baral, 2023). Therefore, the following hypotheses are proposed.

*H1a*: Perceived gamification directly and positively influences soccer learning engagement.

*H1b*: Perceived gamification indirectly but positively influence soccer learning engagement through flow experience.

*H1c*: Perceived gamification indirectly but positively influence soccer learning engagement through mental toughness.

*H1d*: Perceived gamification indirectly but positively influence soccer learning engagement through the combined effect of flow experience and mental toughness.

Perceived self-control (PSC) is learners’ perceived ability to maintain attention, suppress impulses, and regulate behavior during training (Tangney et al., 2004; Baumeister et al., 2007). In gamified XR soccer instruction, PSC provides the self-regulatory capacity needed to remain task-focused and persist through graded challenges (Lu, 2023), thereby increasing the likelihood that learners can enter and sustain a flow experience characterized by clear goals and immediate feedback (Csikszentmihalyi, 1990). While XR can present calibrated difficulty and feedback cues, these affordances do not automatically yield flow unless learners can allocate attention, resist off-task impulses, and regulate pacing across repeated practice episodes (Duckworth and Seligman, 2005). Therefore, PSC is theorized as a proximal individual resource that helps learners capitalize on XR-supported challenge–skill balance and feedback salience, strengthening flow experience and, in turn, learning engagement. Utilizing gamified learning platforms to enhance students’ ability to learn soccer autonomously can help them adapt to an autonomous learning environment. This facilitates better acceptance of soccer teaching content (Lyu and Park, 2025). Therefore, the following hypotheses are proposed.

*H2a*: Perceived self-control directly and positively influences soccer learning engagement.

*H2b*: Perceived self-control indirectly but positively influence soccer learning engagement through flow experience.

*H2c*: Perceived self-control indirectly but positively influence soccer learning engagement through mental toughness.

*H2d*: Perceived self-control indirectly but positively influence soccer learning engagement through the combined effect of flow experience and mental toughness.

A flow experience (FE) is a state of deep concentration with clear goals and immediate feedback. It is accompanied by a loss of self-awareness and an altered perception of time (Csikszentmihalyi, 1990; Jackson and Eklund, 2002). This state occurs when skills and challenges are balanced and feedback is immediate. XR’s high presence and precise telemetry algorithms can manipulate these states. In physical education, meta-analytic and systematic evidence links flow to improved sports performance and persistence (Harris et al., 2023), which supports its role as a proximal driver of SLE and SA. In sports, FE can improve decision-making speed, technical execution, and persistence, thus enhancing learning engagement (Goh and Yang, 2021).

Meanwhile, adaptive XR soccer instruction fosters frequent FE by maintaining optimal challenge levels. These episodes serve as structured micro-adversities, strengthening mental toughness (MT) through repeated emotional regulation and resource consolidation (Gucciardi et al., 2015). Consequently, FE provides a proximal pathway for MT development. Furthermore, MT mediates the relationship between FE-related commitment and learning engagement (St Clair-Thompson and Devine, 2023). The connection between FE and MT represents the shift from “doing” to “being.” Frequent FE episodes serve as structured micro-adversities. Conceptually, the flow stage is rich in mastery experiences, which can provide a pathway from FE to MT through sequential mediation by accumulating efficacy and coping resources. Therefore, the following hypotheses are proposed.

*H3a*: Flow experience directly and positively influences soccer learning engagement.

*H3b*: Flow experience indirectly but positively influence soccer learning engagement through mental toughness.

Mental toughness (MT) is a state-like psychological resource. Its purposefulness, flexibility, and efficiency enable the formulation and maintenance of goal-oriented pursuits (Gucciardi, 2017). Related research has consistently identified MT as one of the most important psychological characteristics related to performance in sports and learning (Crust, 2007). MT represents commitment under pressure, which requires control as a foundation (Liew et al., 2019). These traits contribute to maintaining consistent performance and recovering from setbacks (Gucciardi, 2017). Previous studies (Gucciardi et al., 2015; Covington and Omelich, 1988; Raymond, 2009) have emphasized the importance of considering the dynamic interactions among cognitive, emotional, and motivational domains when conceptualizing MT-related processes. Therefore, gamified XR-related soccer instruction, with its opportunities for graded exposure, safe failure, and repeated mastery, can provide micro-adversities and successes, thus potentially building mental toughness over time. Thus, the following hypotheses are proposed.

*H4*: Mental toughness directly and positively influences soccer learning engagement.

Soccer learning engagement (SLE) is defined as the investment of time and attention, as well as the display of continuous learning behaviors related to skill acquisition (Morgans et al., 2014; Ericsson et al., 1993). SLE indicators measure behavioral, cognitive, and emotional investments in soccer learning. Furthermore, emotional investment brought about by digital technology can reinforce users’ behaviors (Wang et al., 2026). Repeated, meaningful investment can strengthen competence, self-goals, and social connections, deepening the sense of identity and attachment to the sport (Funk and James, 2001).

Sports attachment occurs when students develop an identity with and dependence on soccer due to the influence of learning soccer in an XR environment. This attachment likely determines how they explore the entire sports and learning system (Moss and St-Laurent, 2001). While Bowlby’s (1979) original attachment theory focused on interpersonal bonds between child and caregiver, it is adapted here to the sporting context. In technology-mediated physical education, the XR environment and the sport itself act as a “secure base.” When the XR system provides consistent feedback (competence) and autonomy, the learner feels secure enough to explore complex skills. This psychological safety fosters an affective bond, sport attachment, similar to the emotional security described in traditional attachment theory, which drives long-term adherence and participation beyond the classroom. Exploring sports situations in qualitatively different ways affects the mental representation of attachment (Carr, 2009). Soccer provide individuals with opportunities to explore and develop their cognitive, physical, and social selves. The achievement of goals in sports has been shown to influence this pattern of exploration (Elliot and McGregor, 2001). Therefore, the following hypotheses are proposed.

*H5*: Flow experience directly positively influence sports attachment.

*H6*: Mental toughness directly positively influence sports attachment.

*H7*: Soccer learning engagement directly influence sports attachment.

### 2.3 Research model construction

**Figure 1:**
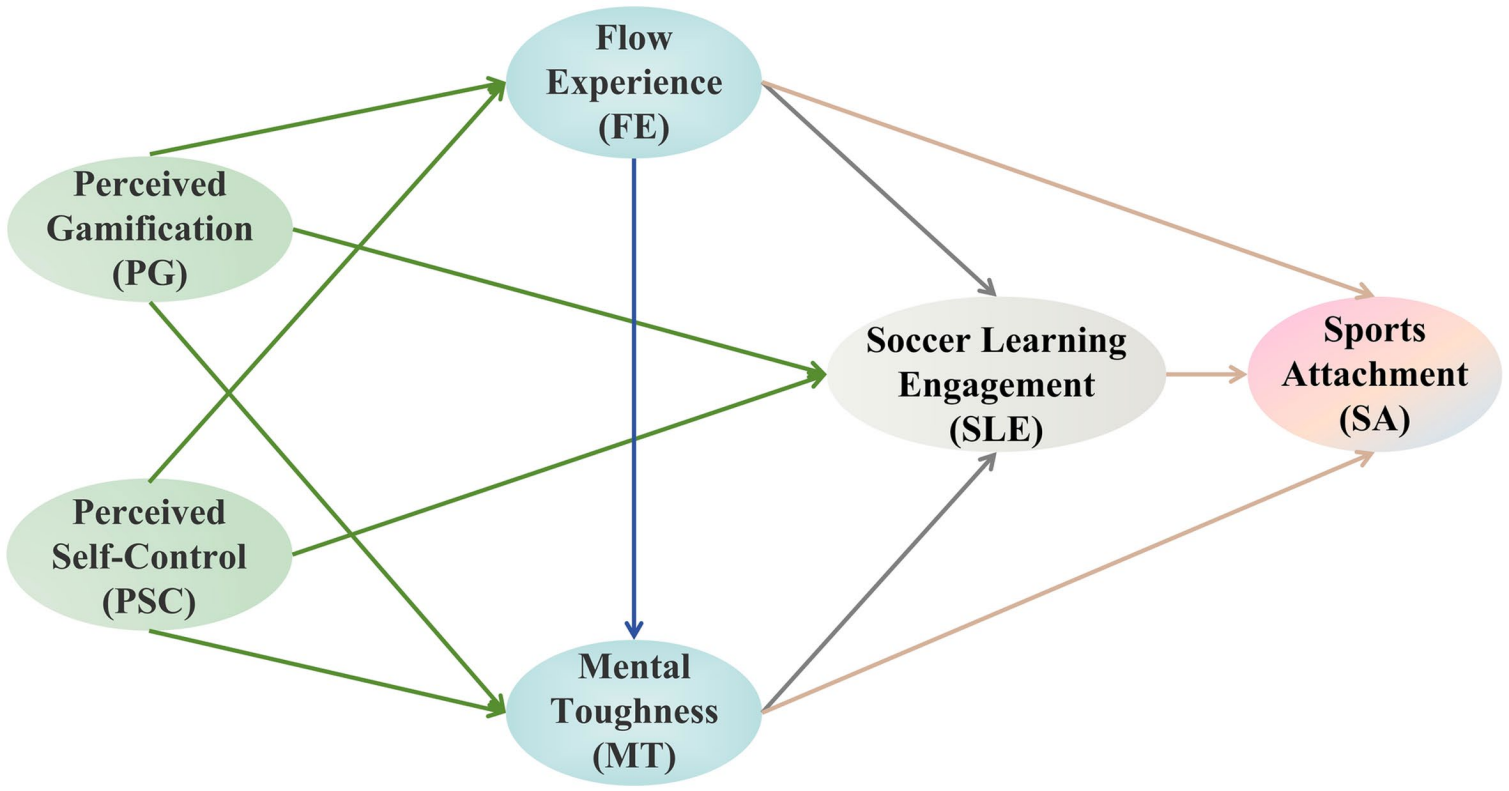
Research model.

Previous studies have typically regarded gamification as a unified approach, failing to specify the mechanisms that generate optimal experiences. This has resulted in inconsistent direct impacts on engagement. Flow and mental toughness were often examined separately rather than as interacting processes, obscuring how transient optimal states could develop lasting resources. To address these issues, this study created a research model (Figure 1) based on the aforementioned hypothesized framework. The model advances theory by distinguishing between the situational optimal state (flow) and the enduring self-regulatory trait (mental toughness), showing how the former facilitates the development of the latter.

The model advances theoretical development by linking need-supportive design and challenge calibration into a single, comprehensive explanation of extended reality (XR) soccer learning (Makransky and Petersen, 2021). This study clarifies the trajectory of the gamification effect, demonstrating a fully mediated path from perceived gamification to learning engagement (Zeng et al., 2024). In addition, the study identifies a continuous path through which the experience of flow consolidates into mental toughness. This refines the distinction between the optimal situational state in teaching and the enduring self-regulatory ability (Hsieh et al., 2024). Finally, the study links learning engagement and investment to sports attachment. This extends the logic of sports socialization to technology-based courses with the explicit educational goal of fostering sports attachment (Nite and Edwards, 2021).

This model aligns with the available evidence in the XR context. In education and sports, XR evaluations and trials have shown that interactive, adaptive tasks improve skill-related outcomes (Darwish et al., 2023; Doolani et al., 2020). This strengthens the rationale of the motivation-behavior association in the model.

## 3 Methodology

### 3.1 Questionnaire design

This study uses a quantitative, survey-based approach to empirically evaluate the proposed research model. Since the necessary data are unavailable in public databases, original data must be collected. The measurement scales were derived from existing literature and adapted based on the context of gamified, XR-related soccer education (see Table 1). The scale consists of 32 items. Previous research (MacKenzie et al., 2011) has shown that formative measurement can fully represent higher-order constructs. To capture the relative importance of these dimensions, all items are measured using a five-point Likert scale ranging from “strongly disagree” (1) to “strongly agree” (5). The adaptation process combined content validation and linguistic procedures (expert review, pilot testing, and bilingual translation/back-translation) to ensure that the items retained the conceptual meaning of the original scales while being appropriate for XR-based soccer instruction.

**Table 1:** Modified scale measures.

Measure	Reference scale	Scale items	
Perceived gamification (PG)	Fitz-Walter et al. (2017) Luo (2023)	PG1	I experienced the joy of challenges
		PG2	I felt the excitement of competition
		PG3	I used game elements to express my unique identity
		PG4	It was convenient for me to communicate and interact with classmates during learning
		PG5	The system’s rewards made me more motivated
Perceived self-control (PSC)	Baumeister et al. (2007)	PSC1	I regulate the pace of my learning on my own
		PSC2	I can promptly correct my mistakes
		PSC3	I can maintain my attention
		PSC4	I can choose appropriate modules and plans
		PSC5	I can effectively regulate my behavior
flow Experience (FE)	Jackson and Eklund (2002)	FE1	I felt that time flew by during my study
		FE2	I was often immersed in the learning process
		FE3	My attention was completely captivated
		FE4	This process awakened my intrinsic interest in soccer learning
		FE5	This process stimulated my curiosity about soccer learning
Mental toughness (MT)	Gucciardi (2017) Gucciardi et al. (2015)	MT1	I am adaptable
		MT2	I have the ability to resist stress
		MT3	I will practice soccer more diligently
		MT4	I always maintain enthusiasm and perseverance
		MT5	I have the confidence to complete challenges
Soccer learning engagement (SLE)	Duda (1998)	SLE1	I am more engaged in learning soccer through XR
		SLE2	I enjoy learning soccer via XR
		SLE3	I will actively seek feedback from the XR system
		SLE4	I will complete the tasks in the XR system on time
		SLE5	My soccer abilities and skills have improved
		SLE6	My performance in class has become more positive
Sports attachment	Bartholomew and Moretti (2002) Carr (2009)	SA1	I have become dependent on the XR-based soccer training mode
		SA2	I have developed an attachment to soccer
		SA3	I have become attached to the XR-based learning mode
		SA4	I think I can explore other sports in the same way
		SA5	I believe that other gamified methods can be incorporated into my soccer learning
		SA6	Without soccer, I feel like I’ve lost myself

At the beginning of the questionnaire, detailed examples of XR applications in soccer learning were provided. Then, the first part of the questionnaire asked respondents about their demographic characteristics. The second part required them to answer questions about all the variables. To ensure high-quality cross-cultural adaptation and semantic validation, we followed Brislin’s (1970) back-translation model. Because the original scales were developed in English, a bilingual sports-education researcher first translated all items into the local language, after which an independent bilingual expert back-translated them into English. Discrepancies were resolved by consensus to achieve semantic and conceptual equivalence before finalizing the wording. To ensure a wide range of respondents and accurate linguistic adaptation, this bilingual procedure was followed before the questionnaire was written and distributed in English.

Content validity was determined through a formal expert panel review. Four experts were selected based on the following criteria: (1) holding a doctoral degree in physical education or teaching technology; (2) having at least five years of experience in XR implementation or sports psychology; (3) having published peer-reviewed papers in these fields. These experts used the content validity index (CVI) to evaluate the clarity, relevance, and representativeness of each item. After making revisions according to the experts’ opinions, the questionnaire was finalized.

Before the main study, a pilot test (*N* = 60) was conducted to evaluate the instrument’s psychometric properties. Internal consistency was confirmed with Cronbach’s alpha values ranging from 0.82 to 0.91 for all constructs. exploratory factor analysis (EFA) demonstrated that all items loaded onto their respective factors (>0.60) with no significant cross-loadings, indicating acceptable preliminary validity. The pre-test results were not included in the total sample size.

### 3.2 Data collection and general demographics

Consistent with established research methods, the hypotheses in this study were tested using survey data. To capture a representative range of experiences, the inclusion criterion “XR-integrated football teaching” was strictly defined as participation in courses in which at least 30% of the teaching time involved skills training using immersive XR devices. Over a three-month period, researchers recruited respondents through university forums and social media pages created specifically for this study, focusing on universities offering XR-integrated physical education courses. While this resulted in a convenience sample, we addressed institutional representativeness by targeting students from 15 different universities across various regions. XR technology in education is evolving rapidly. Therefore, respondents had to be students over 18 years old who had experienced XR-integrated soccer teaching within the past two years, as a more distant technological experience may be completely different from the present.

Of the 700 completed questionnaires initially collected, 80 were excluded due to incompleteness or invalidity (e.g., two questionnaires had the same IP address, and the completion time for some was less than half the average time). Thus, the final sample used for structural validation and hypothesis testing consisted of 620 valid questionnaires, with a qualification rate of 88.57%. Accordingly, the sampling frame represents higher education institutions that have already adopted XR-integrated soccer instruction. The results should therefore be generalized to comparable XR-adopting settings rather than to the full population of universities or sports.

Of the 620 samples, males slightly outnumbered females (57.1% male and 42.9% female). The age distribution was concentrated in the traditional college student age range: 65.0% were aged 18–25, 25.0% were aged 26–29, and 10.0% were aged 30 and over. Undergraduates accounted for the largest proportion (42.1%), followed by postgraduate students (36.0%). On average, XR courses accounted for a high percentage of all soccer learning courses. Eighty-six percent of respondents reported that XR was integrated into more than 40% of soccer courses, and 62.9% reported that XR was integrated into more than 60% of soccer courses. The average proportion of time spent on soccer among all sports also showed a similar concentration. Seventy-seven point 9% of respondents reported that soccer accounted for more than 40% of all sports, and 51.0% reported that it accounted for more than 60%. These results suggest that respondents have recently engaged meaningfully with XR-integrated soccer pedagogy, which supports this study’s focus on contemporary implementation. Notably, Kline and Santor (1999) recommended that the sample size be at least 10 times the number of measurement items. The final sample exceeded the general adequacy criterion for a structural model with 32 indicators (≥320 observations), thereby ensuring sufficient statistical power for the PLS-SEM analysis (Yu et al., 2025).

### 3.3 Analysis method

Kline and Santor (1999) suggested that the sample size should be at least 10 times the number of measurement items. Since the six variables in this study involve 32 measurement items, the sample size available for analysis should be greater than 320 to support the statistical power of subsequent analyses. The 620 valid samples in this study provide strong power and precision for structural estimation and hypothesis testing (Yu et al., 2025).

This study selects structural equation modeling (SEM) for data modeling and analysis. SEM estimates relationships among interrelated variables while accounting for measurement errors. There are two approaches, including covariance-based structural equation modeling (CB-SEM) and partial least squares-structural equation modeling (PLS-SEM). CB-SEM focuses on theory validation, while PLS-SEM uses a causal-predictive approach to explain the variance of the dependent variable (Chin et al., 2020). PLS-SEM is highly effective for studies with small sample sizes, non-normal data distributions, and complex models involving intricate interdependencies among structures (Hair et al., 2021). Its variance-based approach addresses the limitations of traditional CB-SEM techniques, providing robust analysis even when sample size and data normality are restricted (Hair et al., 2019). In addition, PLS-SEM can simultaneously accommodate both formative and reflective concepts, ensuring an overall evaluation of the model.

PLS-SEM was adopted as the primary validation approach owing to its suitability for early-stage theory development (Shiau et al., 2019) and its emphasis on predictive assessment (Benitez et al., 2020). This method concurrently models latent constructs, accounts for measurement error in exogenous and endogenous variables, and estimates all hypothesized relations within a single framework, thereby attenuating statistical bias and diminishing the risk of inferential error. Following the two-step procedure (Hair et al., 2021), we first evaluated the measurement model using SPSS and SmartPLS, and subsequently estimated and tested the structural paths.

## 4 Results

In PLS-SEM, the measurement and structural models are assessed jointly, and subsequent analyses proceed only when both satisfy established evaluation criteria (Anderson and Gerbing, 1988).

### 4.1 Measurement model evaluation

The PLS-SEM measurement procedures include examining empirical dimensionality with principal component analysis, evaluating reliability and convergent validity with Cronbach’s *α* values, composite reliability, and average variance extracted, and assessing discriminant validity with Fornell–Larcker criteria and HTMT ratios. These procedures determine construct quality before the structural assessment.

First, Harman’s single-factor approach and a partial least squares (PLS)-based collinearity surrogate model were adopted to assess common method bias. An unrotated principal component analysis of all 32 indicators (Maćkiewicz and Ratajczak, 1993) yielded six components with eigenvalues greater than 1. Collectively, these components explained 76.91% of the variance. The first unrotated component accounted for only 32.21%. This pattern indicates that the variance was not dominated by a single underlying factor attributable to the common method. The full collinearity diagnosis of the structural model further supports this finding. The variance inflation factors in the endogenous structure ranged from 1.109 to 1.326, which is far below the conservative benchmark of 3.3 (Hair, 2014). Taken together, common method bias is unlikely to significantly distort the estimated relationships, which provides a preliminary basis for the structural plausibility of subsequent validation (Benitez et al., 2020).

To provide an additional, more stringent diagnostic, this study also estimated an unmeasured latent method-factor model in which each indicator loaded on both its theoretical construct (*R*_1_) and a common method factor (*R*_2_) (Wang et al., 2025). The substantive loadings remained high, with an average *R*_1_^2^ of 0.765 across items, whereas the method-factor loadings were very small, with an average *R*_2_^2^ of 0.002 and all individual *R*_2_^2^ values below 0.015. This pattern indicates that the shared method factor explains only a negligible proportion of variance beyond the substantive constructs and converges with the Harman and collinearity tests in suggesting that common method variance is unlikely to bias the reported relationships (see Appendix A for details of the CMB test).

Secondly, the reliability and convergent validity of all six constructs (Table 2) were satisfactory in PLS-SEM (Hair, 2014). Cronbach’s alpha values exceeded the adequacy threshold of 0.70. Composite reliability was uniformly high, surpassing the 0.70 benchmark and evidencing strong internal consistency. Average variance extracted (AVE) for each construct was greater than 0.50, supporting convergent validity under conventional criteria. Collectively, these findings indicate that the measurement model possesses adequate psychometric properties for subsequent structural estimation and hypothesis testing within the proposed framework.

**Table 2:** Reliability and validity.

Variable	Cronbach’s alpha	Composite reliability (rho_c)	Average variance extracted (AVE)
Flow experience (FE)	0.945	0.958	0.821
Mental toughness (MT)	0.939	0.954	0.805
Perceived gamification (PG)	0.908	0.932	0.734
Perceived self-control (PSC)	0.909	0.933	0.736
Sports attachment (SA)	0.950	0.960	0.800
Soccer learning engagement (SLE)	0.911	0.931	0.693

Thirdly, the Fornell–Larcker matrix substantiated discriminant validity. Specially, for each construct in Table 3, the square root of its AVE on the diagonal exceeded all inter-construct correlations, thereby satisfying the classical criterion (Fornell and Larcker, 1981). These results were consistent with previously reported AVE estimates, confirming that each latent variable captured more variance from its own indicators than from other constructs. Overall, the results supported the distinctiveness of the constructs and alleviated concerns about multicollinearity in the subsequent PLS-SEM analysis.

**Table 3:** The Fornell–Larcker matrix.

Variable	FE	MT	PG	PSC	SA	SLE
Flow experience (FE)	**0.906**					
Mental toughness (MT)	0.343	**0.897**				
Perceived gamification (PG)	0.406	0.381	**0.857**			
Perceived self-control (PSC)	0.337	0.389	0.314	**0.858**		
Sports attachment (SA)	0.289	0.271	0.301	0.228	**0.894**	
Soccer learning engagement (SLE)	0.229	0.301	0.206	0.313	0.318	**0.832**

The bolded part is the square root of the AVE.

Finally, the HTMT ratios indicated that all pairs of constructs had clear discriminant validity. Table 4 shows that the values ranged from 0.222 to 0.437, well below the conservative benchmark of 0.85 commonly adopted in behavioral research, suggesting that each construct was empirically distinct from the others (Henseler et al., 2015). Combined with the previously reported Fornell–Larcker results, the HTMT evidence supported conducting the structural analysis without taking corrective measures to address construct ambiguity.

**Table 4:** The heterotrait-monotrait (HTMT) ratio.

Variable	FE	MT	PG	PSC	SA	SLE
Flow experience (FE)	—					
Mental toughness (MT)	0.363	—				
Perceived gamification (PG)	0.437	0.412	—			
Perceived self-control (PSC)	0.361	0.419	0.341	—		
Sports attachment (SA)	0.304	0.285	0.323	0.244	—	
Soccer learning engagement (SLE)	0.245	0.321	0.222	0.344	0.333	—

### 4.2 Structural model evaluation and path testing

After validating and verifying the measurement model, the structural model can be evaluated to test the paths and examine the hypotheses. In PLS-SEM, evaluation of the structural model is based on multiple criteria, including the coefficient of determination (*R*^2^), predictive relevance (*Q*^2^), assessment of collinearity, effect size (*f*^2^), and path coefficients.

Specifically, the structural model exhibited a moderate level of explanatory power and predictive relevance (Table 5). The explanatory power of the endogenous variance was weak to moderate. The *R*^2^ values were 0.214 for FE, 0.246 for MT, and lower for sports attachment (SA = 0.166) and soccer learning engagement (SLE = 0.145). The adjusted *R*^2^ had minimal shrinkage, indicating stable estimates (Hair, 2014). Out-of-sample prediction is supported by positive *Q*^2^ predict across all constructs, meeting the criterion whereby values greater than zero indicate predictive relevance. Proximal states (FE and MT) were relatively more predictable than downstream outcomes (SA and SLE) (Shmueli et al., 2019). The error metrics were consistent with this pattern: FE (RMSE = 0.893; MAE = 0.690) and MT (RMSE = 0.886; MAE = 0.689) had the lowest RMSE and MAE respectively, while SA (RMSE = 0.956; MAE = 0.748) and SLE (RMSE = 0.950; MAE = 0.770) had higher RMSE and MAE, respectively. This indicates that the mediating variables had better point predictability than the distal outcomes. Taken together, these metrics suggest that the cross-validated predictive performance of the model is acceptable and that the model’s strongest prediction accuracy is concentrated on the proximal psychological constructs that are most directly specified by design and self-regulatory antecedents (Benitez et al., 2020).

**Table 5:** Explanatory power and predictive performance (*R*^2^, *Q*^2^, RMSE, MAE).

Variable	*R* ^2^	*R*^2^ adjusted	*Q*^2^ predict	RMSE	MAE
Flow experience (FE)	0.214	0.211	0.208	0.893	0.69
Mental toughness (MT)	0.246	0.242	0.221	0.886	0.689
Sports attachment (SA)	0.166	0.162	0.092	0.956	0.748
Soccer learning engagement (SLE)	0.145	0.139	0.103	0.95	0.77

Moreover, direct effect path estimations support a mechanism whereby design evaluations stimulate proximal states, which then mobilize engagement and attachment. FE is jointly predicted by PG (*β* = 0.333, *p* < 0.001, *f*^2^ = 0.127) and PSC (*β* = 0.232, *p* < 0.001, *f*^2^ = 0.062), with gamification having a stronger impact. MT is shaped by PSC (*β* = 0.261, *p* < 0.001, *f*^2^ = 0.077) and PG (*β* = 0.235, *p* < 0.001, *f*^2^ = 0.058) and reinforced by FE (*β* = 0.160, *p* < 0.001, *f*^2^ = 0.027), which confirms the link between flow and toughness. Additionally, SLE increases with enhanced perceived self-control (*β* = 0.204, *p* < 0.001, *f*^2^ = 0.038) and toughness (*β* = 0.178, *p* < 0.001, *f*^2^ = 0.028), though FE contributes less (*β* = 0.083, *p* = 0.035, *f*^2^ = 0.006). Taken together, these patterns support H2a, H4a and H3. Conversely, the direct path from PG to SLE is not supported (*β* = 0.041, *p* = 0.390), which does not support H1a and suggests that gamification operates via mediating factors rather than directly. SA is promoted by SLE (*β* = 0.234, *p* < 0.001, *f*^2^ = 0.058), FE (*β* = 0.189, *p* < 0.001, *f*^2^ = 0.037) and MT (*β* = 0.135, *p* = 0.001, *f*^2^ = 0.018), thus establishing behavioral and experiential pathways to attachment. These effects support H5, H6 and H7. Notably, the confidence intervals of all significant paths do not contain zero, and multicollinearity is negligible with a VIF ranging from 1.109 to 1.326. This is well below the common threshold for PLS-SEM (Hair et al., 2021) (see Table 6).

**Table 6:** Test for direct effects.

Hypothesis	Direct effect path	Beta	SD	*T*	*p*	Lower	Upper	*f* ^2^	VIF	Result
—	PG → FE	0.333	0.036	9.240	0.000	0.262	0.403	0.127	1.109	Supported
—	PSC → FE	0.232	0.037	6.356	0.000	0.158	0.302	0.062	1.109	Supported
—	PG → MT	0.235	0.033	7.087	0.000	0.167	0.298	0.058	1.25	Supported
—	PSC → MT	0.261	0.036	7.322	0.000	0.192	0.333	0.077	1.178	Supported
—	FE → MT	0.160	0.037	4.292	0.000	0.088	0.231	0.027	1.272	Supported
H1a	PG → SLE	0.041	0.048	0.860	0.390	−0.053	0.132	0.001	1.323	Not supported
H2a	PSC → SLE	0.204	0.045	4.552	0.000	0.113	0.290	0.038	1.268	Supported
H3	FE → SLE	0.083	0.039	2.107	0.035	0.005	0.159	0.006	1.306	Supported
H4a	MT → SLE	0.178	0.044	4.061	0.000	0.090	0.263	0.028	1.326	Supported
H5	FE → SA	0.189	0.040	4.700	0.000	0.106	0.265	0.037	1.156	Supported
H6	MT → SA	0.135	0.041	3.327	0.001	0.055	0.215	0.018	1.205	Supported
H7	SLE → SA	0.234	0.048	4.864	0.000	0.138	0.325	0.058	1.122	Supported

Furthermore, one of the objectives of this study was to explore the mediating roles of FE and MT in the relationships between PG, PSC, and SLE. Following the suggestions of Preacher and Hayes (2008), the bootstrapping method was employed to test the indirect effects during the process of testing the mediation hypotheses. The significance of the indicator weights was tested by evaluating the *t-*values. At a 5% significance level, an indicator weight is considered statistically significant if its *t-*value is greater than 1.960 (two-tailed test).

The mediation effect tests in Table 7 indicate that PG exerts its influence on SLE exclusively through psychological pathways. The indirect effects through FE (*β* = 0.028, *p* = 0.040), MT (*β* = 0.042, *p* < 0.001), and the chained mediation through FE and MT (*β* = 0.009, *p* = 0.007) are all significant, supporting H1b, H1c, and H1d, while the direct path from PG to SLE is not significant. This suggests that flow and toughness achieve full mediation. In contrast, PSC demonstrates a distinct pattern. The indirect effect through MT is found to be significant (*β* = 0.046, *p* < 0.001), supporting H2c, and the serial path through FE and MT is also found to be significant (*β* = 0.007, *p* = 0.012), supporting H2d. However, the path through FE alone is not found to be significant (*β* = 0.019, *p* = 0.054), not supporting H2b. In view of the identified direct effect of PSC on SLE, this configuration is consistent with the complementary partial mediation effect, meaning that self-control can mobilize engagement both directly and through toughness.

**Table 7:** Test for mediating effects.

Hypothesis	Indirect effect path	Beta	SD	*T*	*p*	Lower	Upper	Result
H1b	PG → FE → SLE	0.028	0.013	2.059	0.040	0.002	0.054	Supported
H1c	PG → MT → SLE	0.042	0.012	3.574	0.000	0.021	0.067	Supported
H1d	PG → FE → MT → SLE	0.009	0.003	2.720	0.007	0.004	0.018	Supported
H2b	PSC → FE → SLE	0.019	0.010	1.928	0.054	0.002	0.041	Not supported
H2c	PSC → MT → SLE	0.046	0.012	3.721	0.000	0.024	0.074	Supported
H2d	PSC → FE → MT → SLE	0.007	0.003	2.517	0.012	0.003	0.013	Supported
H4b	FE → MT → SLE	0.028	0.010	2.848	0.004	0.012	0.052	Supported

The mediation results also demonstrate that FE promotes SLE through a relatively small direct effect and a significant indirect effect via MT (*β* = 0.028, *p* = 0.004), supporting H4b, which confirms that the theoretically postulated link between flow and toughness serves as a channel for guiding behavior. For all supported indirect effects, the confidence intervals do not contain zero, which supports their reliability under the bootstrapped PLS-SEM estimates (Preacher and Hayes, 2008).

**Figure 2:**
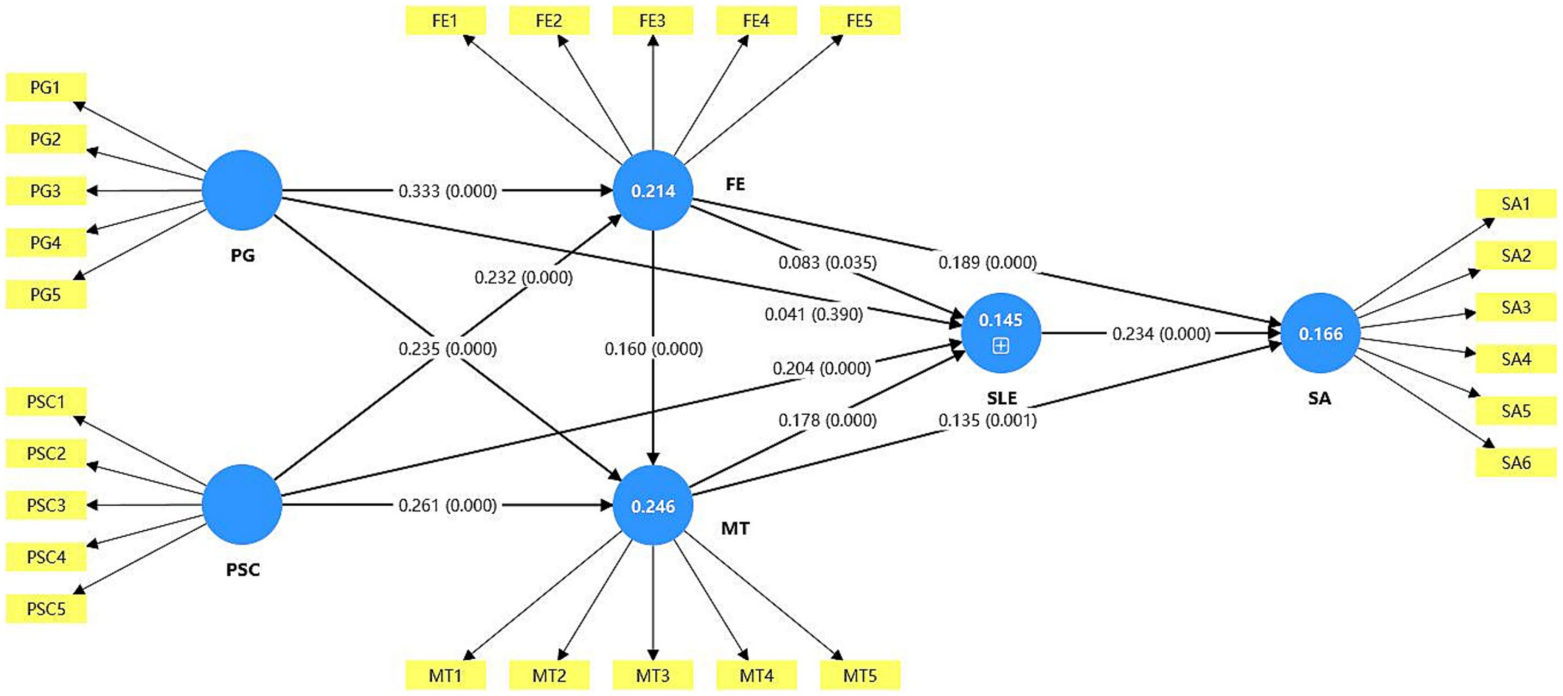
PLS-SEM testing results.

Concurrently, the estimation results of the relationships among constructs in the structural model are summarized through Figure 2. PSC is a direct and indirect driver of engagement, while PG is an indirect driver through FE and MT. Additionally, a clear path linking engagement and attachment is evident.

The structural model results show that perceived gamification and perceived self-control significantly predict flow experience, while flow and mental toughness contribute to sports attachment. The model explains 14.5% of the variance in soccer learning engagement (SLE; *R*^2^ = 0.145) and 16.6% of the variance in sports attachment (SA; *R*^2^ = 0.166). These *R*^2^ values are modest, indicating that the psychological mechanisms captured by the model account for a limited but non-trivial portion of the variability in students’ engagement and attachment. Rather than suggesting that SLE and SA are predominantly determined by flow-related processes, these results imply that substantial variance is likely driven by other contextual, interpersonal, and dispositional factors that fall outside the scope of the present model. The path from flow experience to SLE is positive but small (*β* = 0.083), which suggests that, although flow contributes to engagement, its unique incremental effect is relatively weak once other predictors are taken into account.

## 5 Discussion

This study examined how perceived gamification and self-control are associated with soccer learning engagement through the mediating roles of flow experience and mental toughness and how these factors relate to sports attachment. The research findings largely support the hypothesized model, clarifying correlational pathways through which gamification and self-control may be linked to deeper engagement and attachment to sports. The findings were interpreted within the extant literature and theoretical frameworks, with emphasis on their incremental contributions to understanding sports attachment and the dynamic interplay between flow and mental toughness. Importantly, given the cross-sectional, self-report design, the results should be interpreted as theory-consistent associations rather than evidence of temporal or causal effects.

In sport and physical education, perceived gamification (PG) is widely regarded as a lever for enhancing participation and motivation. However, in this study, PG did not exert a direct effect on soccer learning engagement, indicating that the mere inclusion of game elements does not automatically elevate engagement. This result diverges from Johnson’s (2016) observations. Instead, PG operated through psychological mediators; that is, effective gamification may necessitate the cultivation of optimal experiences to yield higher engagement, consistent with existing research on gamification and flow (Cai et al., 2024; Zhang et al., 2025). Moreover, the intrinsically appealing and rewarding nature of gamified soccer training may be linked to persistence through challenge and may co-occur with a more resilient mindset, thereby strengthening motivation. The observed sequential mediation via flow and mental toughness further suggests a theory-aligned coupling between these processes in gamified learning, although the present data cannot establish temporal ordering among these constructs.

Perceived self-control (PSC) demonstrated a direct, positive association with soccer learning engagement, underscoring the importance of learners’ willpower and self-regulatory restraint in physical education. This pattern aligns with broader psychological evidence (Tangney et al., 2018). The mediating effect of mental toughness (MT) is also supported, suggesting that PSC is associated with MT. Specifically, students with greater self-discipline may confront training challenges more actively and build confidence and coping abilities over time. This enhanced MT is, in turn, linked to higher engagement. In contrast, flow does not produce a direct mediating effect. This is mainly because PSC reflects effortful, top-down regulation, which enhances self-monitoring and cognitive load. These conditions may be less conducive to the formation of self-actualized flow (Tangney et al., 2018). Transient flow experience (FE) provides mastery cues that may broaden and build resilience resources, thereby relating to MT (Schimschal et al., 2022; Salanova et al., 2006).

However, it is important to reflect on the interpretive limitations here. Self-reported PSC may be subject to social desirability bias, where students over-report their discipline. The sequential path of PSC-FE-MT-SLE suggests an associational pathway, but the data cannot confirm that flow precedes mental toughness in a temporal sense.

Apart from serving as mediating factors, flow experience (FE) and mental toughness (MT) independently influence soccer learning engagement. When learners are in a flow state, they are highly engaged in the present moment, which is consistent with existing research (Hamari et al., 2016). Educators and coaches often try to create conditions that stimulate FE in order to encourage students’ sustained participation and progress, thereby forming a positive motivation cycle. However, the smaller coefficient represents a limited linear impact on SLE. Future research should examine whether flow exerts stronger effects on more fine-grained indicators and whether non-linear or interaction effects better capture its role in XR-based sport education. Moreover, MT also significantly impacts engagement directly, demonstrating that learners’ MT and determination can directly translate into higher engagement in physical education. These results align with prior evidence linking MT to sustained, high-quality training behaviors and goal persistence (Cormier et al., 2025; St Clair-Thompson et al., 2015). Accordingly, within XR-integrated soccer pedagogy, the direct effects of FE and MT underscore the centrality of optimal psychological states and traits for eliciting engagement. At the same time, the observed effect sizes should be interpreted in light of measurement constraints and shared-method assessment, and not as definitive estimates of real-world behavioral impact.

Ultimately, findings for sports attachment (SA) substantiate a motivational progression from experiential states and resilience to behavior and identity formation. In this study, SA encompasses vitality and dedication alongside the internalization of sport as a salient personal identity. Engagement emerged as the strongest predictor of SA, with FE and resilience contributing additional explanatory power. This configuration mirrors the transition from attraction to attachment through repeated, meaningful engagement. This process is described in the psychological continuum model (Funk and James, 2001). It further indicates that, in technology-mediated instruction, designing for optimal experiences and cultivating resilient self-regulatory capacities are complementary strategies for strengthening SA. From an applied perspective, the study’s approach of combining challenges with skills to trigger flow and build personal resources that sustain effort under pressure is a useful model for enhancing engagement and solidifying long-term commitment to sports (Clough et al., 2002). However, because attachment implies longer-term identity development, future studies should test this progression using longitudinal designs and objective participation indicators to verify whether engagement precedes attachment over time.

## 6 Conclusion

This study elucidates how perceived gamification and self-control are linked to engagement in XR-integrated soccer instruction and are associated with attachment to the sport. Specifically, in XR-enabled courses, gamified design renders participation intrinsically enjoyable (flow) and challenges tractable (resilience), while self-control sustains participation even under less favorable conditions. The findings further affirm that engagement is a precursor to attachment. Meanwhile, an engaging learning experience not only enhances immediate skill acquisition but also fosters a durable affective bond with physical activity.

Theoretically, this work advances sports education psychology in three respects. First, it demonstrates that gamification exerts its primary effects through indirect associations with optimal experiential states and resilience. Second, it delineates design-driven and agentic pathways by showing that self-control relates to willpower and resilience. In particular, it identifies a sequential mechanism in which flow experiences are statistically consistent with mastery signals that co-occur with mental toughness, and, in turn, are linked to learning effort. Third, it positions sports attachment as a downstream identity outcome emerging from repeated, meaningful engagement as a conceptual progression that should be tested temporally. Together, these contributions integrate motivational experiences with psychological resources to explain how XR-based physical education may transform learners’ engagement into attachment.

Practically, the results propose a dual-track blueprint for XR-integrated soccer pedagogy. Instructors should align task difficulty with learners’ skills and provide clear, immediate feedback to reliably induce fluent learning states. Concurrently, they should cultivate resilience by incorporating safe failure cycles, graded stressors, reflective debriefings, and regular activities that strengthen perceived control and commitment. Since self-control perception can directly mobilize students’ efforts, XR-based educational tool designs should include pacing tools, progress dashboards, goal-setting prompts, and time-management support. Gamification elements should be closely integrated with diagnostic feedback and adaptive challenges, rather than being used merely as superficial rewards. Monitoring engagement and attachment as core outcomes can guide the iterative calibration of XR scenarios, maintaining meaningful engagement and enhancing students’ identification with the sport.

Beyond psychological pathways, the practical implementation of XR-integrated soccer instruction faces significant institutional hurdles. The high financial costs of VR hardware and software maintenance present a risk of a “digital divide” between well-funded and under-resourced institutions. Furthermore, the success of these models is heavily dependent on teacher expertise. Many physical educators may lack the technical pedagogical content knowledge (TPACK) required to effectively calibrate XR tasks, potentially leading to technical distractions rather than skill acquisition. Therefore, the “perceived gamification” measured this study may be as much a reflection of the instructor’s implementation fidelity as it is of the software itself.

This study offers educators a clear, testable process model and specific design levers to cultivate proactive, resilient, sports-loving learners. Nevertheless, this study has limitations. The cross-sectional self-report PLS-SEM design restricts causal inferences. Specifically, the exclusive reliance on self-reported measures may introduce common method bias, social desirability, and recall effects, and the current scales may not fully represent moment-to-moment flow episodes, behavioral engagement, or context-specific mental toughness under actual performance pressure. Sampling only from universities that have already used XR limits external validity, ignores clustering of courses and teachers, and fails to capture temporal dynamics of the flow-to-resilience sequence. Consequently, the present sample should be viewed as analytically adequate for examining the proposed process model within XR-integrated soccer courses, but not as statistically representative of all university students or of institutions that have not yet implemented XR infrastructure.

Therefore, future research should adopt longitudinal or experimental designs combined with multi-wave measurements, pre-registered analyses, retention validation, and multilevel modeling. Furthermore, from a research context perspective, the scenario involves higher education soccer on heterogeneous XR platforms and teaching styles; thus, the universality across sports, ages, and cultures remains uncertain. Thus, future research should replicate the study in different settings, compare cooperative and competitive gamification, specify platform affordances and implementation fidelity, link psychological pathways to objective educational and engagement outcomes, and test scalable resilience scaffolding and adaptive challenge policies simultaneously in actual courses. In particular, combining platform telemetry with experience sampling can better validate the theorized flow-to-toughness progression and reduce dependence on single-wave self-report data.

## Data Availability

The raw data supporting the conclusions of this article will be made available by the authors, without undue reservation.

## Appendix A

**Table A1:** CMB test results.

Items	*R*_1_ (factor loadings)	*R*_1_^2^	*R*_2_ (CMB factor loadings)	*R*_2_^2^
FE1	0.861^***^	0.741	0.017	0.000
FE2	0.946^***^	0.895	−0.031	0.001
FE3	0.843^***^	0.711	0.024	0.001
FE4	0.954^***^	0.910	−0.025	0.001
FE5	0.922^***^	0.850	0.017	0.000
MT1	0.919^***^	0.845	−0.053^*^	0.003
MT2	0.877^***^	0.769	0.002	0.000
MT3	0.918^***^	0.843	−0.008	0.000
MT4	0.850^***^	0.723	0.048	0.002
MT5	0.921^***^	0.848	0.011	0.000
PG1	0.791^***^	0.626	−0.003	0.000
PG2	0.887^***^	0.787	0.000	0.000
PG3	0.770^***^	0.593	0.042	0.002
PG4	0.853^***^	0.728	0.012	0.000
PG5	0.970^***^	0.941	−0.045^*^	0.002
PSC1	0.729^***^	0.531	0.119^**^	0.014
PSC2	0.888^***^	0.789	0.004	0.000
PSC3	0.808^***^	0.653	−0.047	0.002
PSC4	0.902^***^	0.814	−0.028	0.001
PSC5	0.952^***^	0.906	−0.043^*^	0.002
SA1	0.856^***^	0.733	0.034	0.001
SA2	0.849^***^	0.721	0.011	0.000
SA3	0.941^***^	0.885	−0.050^**^	0.003
SA4	0.883^***^	0.780	0.036	0.001
SA5	0.930^***^	0.865	−0.022	0.000
SA6	0.904^***^	0.817	−0.007	0.000
SLE1	0.714^***^	0.510	0.098^**^	0.010
SLE2	0.926^***^	0.857	−0.123^***^	0.015
SLE3	0.875^***^	0.766	−0.007	0.000
SLE4	0.827^***^	0.684	0.007	0.000
SLE5	0.819^***^	0.671	0.019	0.000
SLE6	0.829^***^	0.687	0.014	0.000
Average		0.765		0.002
