# Correction: Flowing toward toughness: serial mediation of flow and mental toughness in gamified XR soccer instruction

**DOI:** 10.3389/fpsyg.2026.1816734

**Published:** 2026-03-05

**Authors:** Chao Li, Daniel Memmert, Guangliang Sang

**Affiliations:** 1 Faculty of Education Science & Technology, Universiti Teknologi Malaysia, Johor Bahru, Malaysia; 2 Institute of Exercise Training and Sport Informatics, German Sport University Cologne, Cologne, Germany; 3 College of Arts and Physical Education, Kangnam University, Yongin, Republic of Korea; 4 School of Engineering, Korea National University of Transportation, Chungju, Republic of Korea

**Keywords:** extended reality (XR), flow experience, gamification, learning engagement, mental toughness, serial mediation, sport attachment

Affiliation 2 “Institute of Exercise Training and Sport Informatics, German Sport University Cologne, Cologne, Germany” was omitted for author Daniel Memmert. This affiliation has now been added for author Daniel Memmert.

Author Daniel Memmert was erroneously assigned to affiliation 1 “Faculty of Education Science & Technology, Universiti Teknologi Malaysia, Johor Bahru, Malaysia.” This affiliation has now been removed for author Daniel Memmert.

The original version of this article has been updated.

